# Relationship between dexamethasone added to periarticular anesthetic infiltration and postoperative nausea and vomiting following total knee arthroplasty under general anesthesia: a retrospective observational study

**DOI:** 10.1186/s40981-020-00372-1

**Published:** 2020-08-21

**Authors:** Toshiyuki Yano, Takashi Imaizumi, Hidemi Matsu-ura, Tomoki Takahashi

**Affiliations:** 1 Division of Anesthesia, Kumamoto Kinoh Hospital, 6-8-1 Yamamuro, Kita-ku, Kumamoto, 860-8518 Japan; 2 Department of Pharmacy, Kumamoto Kinoh Hospital, 6-8-1 Yamamuro, Kita-ku, Kumamoto, 860-8518 Japan; 3 Department of Orthopedic Surgery, Kumamoto Kinoh Hospital, 6-8-1 Yamamuro, Kita-ku, Kumamoto, 860-8518 Japan

**Keywords:** Glucocorticoids, Multimodal cocktail, Periarticular injection, PONV, Postoperative analgesia, Prosthetic joint infection, Steroids, Surgical site infection

## Abstract

**Background:**

Periarticular anesthetic infiltration (PAI) with a corticosteroid is a modality for pain control following total knee arthroplasty (TKA). Systemic corticosteroids are an established antiemetic for the prophylaxis of postoperative nausea and vomiting (PONV). The purpose of this retrospective observational study was to elucidate the relationship between dexamethasone added to PAI and PONV in patients who underwent TKA.

**Methods:**

Data from 435 patients who received PAI using ropivacaine with or without dexamethasone were reviewed. The primary outcome was the incidence of PONV within 24 h following TKA. The incidence of deep incisional and organ/space surgical site infection (SSI) within the first year was also assessed.

**Results:**

The overall incidence of PONV was 23.2%. A multivariate logistic regression analysis showed that dexamethasone added to PAI was independently associated with a reduced incidence of PONV (adjusted odds ratio, 0.23; 95% confidence interval, 0.12–0.44, *P* < 0.001). The incidence of PONV and rescue analgesic requirements within 24 h were lower in patients who received PAI with dexamethasone than in those who received PAI alone (19.5% vs 49.1%, *P* < 0.001, 7.9% vs 29.1%, *P* < 0.001, respectively). SSI developed in one out of the 55 patients who received PAI alone, but in none of those who received PAI with dexamethasone.

**Conclusions:**

Dexamethasone added to PAI for postoperative pain management was independently associated with a lower risk of PONV within 24 h of TKA.

## Background

Total knee arthroplasty (TKA) is an effective and reliable procedure for relieving pain and improving knee joint function in patients with end-stage osteoarthritis of the knee. TKA causes severe pain immediately after surgery and postoperative pain control remains a challenging issue. Periarticular anesthetic infiltration (PAI) with an α-adrenoceptor agonist, opioid, non-steroidal anti-inflammatory drug (NSAID), or corticosteroid is a modality for achieving similar pain relief to epidural blocks and peripheral nerve blocks [1, 2]. Local corticosteroids have been shown to act as an adjuvant for local anesthetics in PAI [3, 4].

Postoperative nausea and vomiting (PONV) is still a common complication in patients and hinders their recovery after surgery. Systemic corticosteroids exert antiemetic effects and are used for the prophylaxis of PONV. However, the prophylactic effects of local corticosteroids on PONV are controversial [4–6]. We designed a retrospective observational study to elucidate the relationship between dexamethasone added to PAI and PONV following TKA using a multivariate analysis. We also investigated the effects of dexamethasone added to PAI on surgical site infection (SSI) because corticosteroids have been implicated in prosthetic joint infection (PJI), which is a rare, but devastating side effect.

## Methods

The Institutional Review Board approved this single-center retrospective observational study and did not require informed consent due to the nature of the study (approval number: JMC270-1831). We selected all adult patients who underwent elective unilateral TKA under general anesthesia and received intraoperative PAI with or without dexamethasone in Kumamoto Kinoh Hospital between September 1, 2015, and October 31, 2017. Although PAI using ropivacaine with dexamethasone was performed in principle during this period, some attending surgeons eliminated dexamethasone from the cocktail solution according to their discretion. Each patient who underwent bilateral TKA separately during this period was treated for a total of two patients. Patients who received a preoperative corticosteroid treatment were excluded from the present study.

All patients were fasted overnight, dehydrated for at least 2 h preoperatively, and premedication with a histamine H_2_ receptor antagonist or proton pump inhibitor was orally administered 2 to 3 h prior to anesthesia. Anesthesia was induced with propofol and maintained with either propofol alone or sevoflurane with/without propofol in combination with remifentanil. Nitrous oxide was not used in the present study. The solution for PAI without dexamethasone consisted of 20 ml of 0.75% ropivacaine and 30 ml of physiological saline, and the solution for PAI with dexamethasone consisted of 20 ml of 0.75% ropivacaine, 2 ml of 0.33% dexamethasone, and 28 ml of physiological saline. Either solution was injected around the periarticular tissues immediately before the application of cement to the cut bone surface. A pneumatic tourniquet was used immediately before implant cementation to wound closure and a surgical vacuum drain was placed before wound closure. Postoperative pain was routinely managed by oral NSAIDs, acetaminophen, or tramadol in combination with metoclopramide for the prophylaxis of nausea by tramadol after the initiation of postoperative oral rehydration, which was withheld for at least 4 h after surgery. Intravenous pentazocine or diclofenac suppositories were administered as a rescue analgesic for intolerable pain.

PONV was defined as either nausea, vomiting, or retching. Episodes of PONV, which were one of the first priority items for the postoperative assessment and routinely recorded by ward nurses, were collected from the nursing records up to 24 h after transferal to the recovery room from the operating room. Patient characteristics including age, sex, weight, height, body mass index, a previous history of PONV/motion sickness, smoking status, American Society of Anesthesiologists-physical status (ASA-PS), coexisting diabetes mellitus, and insulin treatment were collected from medical records. Anesthesia-related variables including the durations of surgery and anesthesia; intraoperative use of inhalational anesthetics, propofol, opioids, and NSAIDs/acetaminophen; and intra-operative fluid balance were extracted from anesthesia records. Postoperative management-related variables including the duration of postoperative supplemental oxygen, postoperative use of oral opioids and antiemetics, and rescue antiemetics and analgesics were collected from medical and nursing records. Patients who developed deep incisional or organ/space SSI, as defined by the Centers for Disease Control and Prevention/National Healthcare Safety Network guidelines [7], within the first year following TKA, were extracted from the institutional SSI registry database.

The sample size was calculated for a multivariate logistic regression analysis according to the rule that outcome events need to be at least ten per each independent variable. In the present study, to achieve the robust predictive performance of the logistic model with ten or fewer predictive variables under the estimated 25% incidence of PONV, we needed at least 400 patients.

The primary outcome was the incidence of PONV within 24 h of TKA. The incidence of deep incisional and organ/space SSI within the first year following TKA was also assessed. Variables were reported as medians with interquartile ranges or absolute values and proportions (%) and were compared using the Wilcoxon rank sum test or Fisher’s exact test. *P* values of < 0.05 were considered to be significant. Logistic regression models were used to elucidate the relationship between dexamethasone added to PAI and the incidence of PONV. The selection of variables was based on previous literature and included female sex, a history of PONV/motion sickness, non-smoking status, duration of anesthesia, use of inhalational anesthetics, and postoperative use of opioids [8]. Variables in patient characteristics according to PONV were also included in the logistic regression model if the significance of differences was *P* < 0.10 for the primary outcome in the univariate analysis. A forced entry multivariate logistic regression analysis was performed to adjust for potential confounders. Adjusted odds ratios (ORs) were reported together with 95% confidence intervals (CIs). All analyses were performed with the free statistical software EZR version 1.28 (http://www.jichi.ac.jp/saitama-sct/SaitamaHP.files/statmed.html) [9].

## Results

Four hundred and seventeen patients underwent unilateral TKA and nine underwent bilateral TKA during the investigation period; therefore, a cumulative total of 435 patients were enrolled in the present study.

The overall incidence of PONV within 24 h of TKA was 23.2% (101 patients). Patient characteristics according to the occurrence of PONV are shown in Table 1. No significant differences were observed in age or ASA-PS between patients who did or did not develop PONV. Height was significantly different, whereas weight and the body mass index were similar between the two groups. The percentages of patients who were female, had a previous history of PONV/motion sickness, and were non-smokers were higher in those who developed PONV than in those who did not (88.1% vs 74.0%, *P* = 0.003, 21.8% vs 8.4%, *P* = 0.001, 100% vs 93.1%, *P* = 0.004, respectively). No significant differences were observed in the types of general anesthesia, durations of surgery and anesthesia, intraoperative use of opioids (fentanyl, pethidine, or pentazocine), intraoperative fluid balance, postoperative use of opioids (tramadol or pentazocine), and postoperative duration of supplemental oxygen. The frequencies of PAI with dexamethasone and intraoperative use of droperidol as a prophylactic antiemetic were lower in patients who developed PONV than in those who did not (73.3% vs 91.6%, *P* < 0.001 and 62.4% vs 74.3%, *P* = 0.024, respectively).

**Table 1:** Characteristics of patients with and without postoperative nausea and vomiting

Variable	Patients without PONV (*n* = 334)	Patients with PONV (*n* = 101)	*P* value
Age, years	75 [68, 80]	75 [70, 81]	0.117
Female sex	247 (74.0)	89 (88.1)	0.003
Weight, kg	61.3 [54.0, 70.5]	60.4 [50.9, 66.0]	0.055
Height, cm	152.1 [146.5, 158.6]	149.1 [146.0, 154.0]	0.003
Body mass index, kg/m^2^	26.1 [24.0, 29.6]	26.5 [24.2, 29.0]	0.881
ASA-PS			0.249
I	14 (4.2)	1 (1.0)	
II	237 (71.0)	78 (77.2)	
III	83 (24.9)	22 (21.8)	
History of PONV/motion sickness	28 (8.4)	22 (21.8)	0.001
Non-smoking status	311 (93.1)	101 (100.0)	0.004
Duration of surgery, min	109 [97, 120]	107 [98, 123]	0.540
Duration of anesthesia, min	151 [138, 164]	149 [139, 163]	0.992
Types of general anesthesia			0.820
Total intravenous anesthesia	165 (49.4)	48 (47.5)	
Volatile anesthetic (sevoflurane) use	169 (50.6)	53 (52.5)	
Nitrous oxide use	0 (0.0)	0 (0.0)	NA
Intraoperative opioids other than remifentanil	314 (94.0)	100 (99.0)	0.059
Intraoperative antiemetic (droperidol)	248 (74.3)	63 (62.4)	0.024
Intraoperative fluid balance, ml	663 [463, 900]	705 [525, 880]	0.521
PAI with dexamethasone	306 (91.6)	74 (73.3)	< 0.001
Postoperative opioids	283 (84.7)	92 (91.1)	0.137
Duration of supplemental oxygen, h	3.0 [3.0, 3.0]	3.0 [3.0, 3.0]	0.862

Data are presented as absolute (%) or median [interquartile range] values

*ASA-PS* American Society of Anesthesiologists-physical status, *NSAIDs* non-steroidal anti-inflammatory drugs, *PAI* periarticular anesthetic infiltration, *PONV* postoperative nausea and vomiting

Table 2 shows the effects of independent variables from patient characteristics on PONV with a multivariate logistic regression analysis. Variables included female sex, a history of PONV/motion sickness, non-smoking status, duration of anesthesia, use of sevoflurane, postoperative use of opioids, and dexamethasone added to PAI. In addition, intraoperative opioids and droperidol were identified as candidate variables for the multivariate analysis with a significance criterion of *P* < 0.10 in patient characteristics by PONV, as shown in Table 1. This analysis revealed that dexamethasone added to PAI (adjusted OR, 0.23; 95%CI, 0.12–0.44, *P* < 0.001) as well as female sex (adjusted OR 2.07; 95%CI, 1.04–4.13, *P* = 0.039), a previous history of PONV/motion sickness (adjusted OR, 3.60; 95%CI, 1.84–7.05, *P* < 0.001), and intraoperative use of droperidol (adjusted OR, 0.46; 95%CI, 0.27–0.78, *P* = 0.004) was independently associated with PONV.

**Table 2:** Multivariate logistic regression analysis of patient characteristics associated with postoperative nausea and vomiting

Variable	OR [95%CI]	*P* value
Female sex	2.07 [1.04–4.13]	0.039
History of PONV/motion sickness	3.60 [1.84–7.05]	< 0.001
Non-smoking status	1.17e+7 [0.00–∞]	0.983
Duration of anesthesia, min	1.00 [0.99–1.01]	0.498
Sevoflurane	1.16 [0.71–1.89]	0.548
Intraoperative droperidol	0.46 [0.27–0.78]	0.004
Intraoperative opioids	7.16 [0.77–66.80]	0.084
Postoperative opioids	1.50 [0.66–3.39]	0.335
Dexamethasone added to PAI	0.23 [0.12–0.44]	< 0.001

*CI* confidence interval, *OR* odds ratio, *PAI* periarticular anesthetic infiltration, *PONV* postoperative nausea and vomiting

Table 3 shows patient characteristics including the incidence of PONV and SSI according to the intervention with PAI with dexamethasone. No significant differences were observed in age, sex, ASA-PS, a previous history of PONV/motion sickness, and non-smoking status between the two groups. Weight and height differed, whereas the body mass index was similar between the two groups. The percentages of patients with diabetes and insulin treatment were lower in those who received PAI with dexamethasone than in those who received PAI alone (16.6% vs 47.3%, *P* < 0.001 and 0.5% vs 10.9%, *P* < 0.001, respectively). Anesthesia-related variables including the duration of anesthesia, fluid balance, and the frequencies of use of sevoflurane, opioids other than remifentanil, NSAIDs/acetaminophen, and droperidol were not significantly different between the two groups, except for the duration of surgery, which was significantly shorter in patients who received PAI with dexamethasone than in those who received PAI alone (107 min vs 114 min, *P* = 0.014). Postoperatively, the duration of supplemental oxygen and frequencies of use of NSAIDs/acetaminophen and tramadol were similar in both groups. The frequencies of the requirement for rescue analgesics and rescue pentazocine were significantly lower in patients who received PAI with dexamethasone than in those who received PAI alone (7.9% vs 29.1%, *P* < 0.001, 2.9% vs 23.6%, *P* < 0.001, respectively). PONV and the requirement for intravenous metoclopramide as a rescue antiemetic were less frequent in patients who received PAI with dexamethasone than in those who received PAI alone (19.5% vs 49.1%, *P* < 0.001, 6.6% vs 36.4%, *P* < 0.001). Deep incisional or organ/space SSI developed in 1 out of 55 patients who received PAI alone, and in none of the 380 patients who received PAI with dexamethasone, and no significant difference was observed between the groups (*P* = 0.126). The patient who developed SSI, classified as organ/space SSI (joint infection), was non-diabetic and the wound was debrided on postoperative day 17.

**Table 3:** Patient characteristics according to the intervention with the addition of dexamethasone to periarticular anesthetic infiltration

Variable	PAI without dexamethasone (*n* = 55)	PAI with dexamethasone (*n* = 380)	*P* value
Age, years	75 [71, 80]	75 [68, 80]	0.479
Female sex	48 (87.3)	288 (75.8)	0.060
Weight, kg	58.0 [49.8, 65.6]	61.5 [54.0, 70.4]	0.026
Height, cm	147.6 [144.0, 152.4]	151.7 [147.0, 157.8]	0.001
Body mass index, kg/m^2^	25.8 [23.7, 29.2]	26.4 [24.1, 29.5]	0.417
Diabetes mellitus	26 (47.3)	63 (16.6)	< 0.001
Insulin treatment	6 (10.9)	2 (0.5)	< 0.001
ASA-PS			0.289
I	0 (0.0)	15 (3.9)	
II	39 (70.9)	276 (72.6)	
III	16 (29.1)	89 (23.4)	
History of PONV/motion sickness	7 (12.7)	43 (11.3)	0.821
Non-smoking status	51 (92.7)	361 (95.0)	0.514
Duration of surgery, min	114 [104, 120]	107 [96, 120]	0.014
Duration of anesthesia, min	152 [142, 162]	151 [138, 164]	0.370
Sevoflurane use	30 (54.5)	192 (50.5)	0.665
Propofol consumption, mg	602 [200, 733]	633 [200, 814]	0.101
Remifentanil consumption, mg	2.00 [1.80, 2.64]	2.10 [1.74, 2.76]	0.704
Intraoperative opioids other than remifentanil	53 (96.4)	361 (95.0)	1.000
Intraoperative NSAIDs/acetaminophen	42 (76.4)	278 (73.2)	0.744
Intraoperative droperidol	39 (70.9)	272 (71.6)	1.000
Intraoperative fluid balance, ml	760 [513, 980]	665 [478, 885]	0.132
Postoperative NSAIDs/acetaminophen	54 (98.2)	379 (99.7)	0.237
Postoperative tramadol	47 (85.5)	326 (85.8)	1.000
Rescue analgesic	16 (29.1)	30 (7.9)	< 0.001
Pentazocine, intravenous	13 (23.6)	11 (2.9)	< 0.001
Diclofenac, suppository	4 (7.3)	20 (5.3)	0.527
Rescue antiemetic	20 (36.4)	25 (6.6)	< 0.001
Duration of supplemental oxygen, h	3.0 [3.0, 3.0]	3.0 [3.0, 3.0]	0.799
PONV	27 (49.1)	74 (19.5)	< 0.001
Deep incisional or organ/space SSI	1 (1.8)	0 (0.0)	0.126

Data are presented as absolute (%) or median [interquartile range] values

*ASA-PS* American Society of Anesthesiologists-physical status, *NSAIDs* non-steroidal anti-inflammatory drugs, *PAI* periarticular anesthetic infiltration, *PONV* postoperative nausea and vomiting, *SSI* surgical site infection

## Discussion

The principal result of the present study is that dexamethasone added to PAI was independently associated with a reduced incidence of PONV within 24 h postoperatively in patients who underwent TKA under general anesthesia. We also demonstrated that dexamethasone added to PAI did not increase the incidence of deep incisional or organ/space SSI within the first year of TKA.

Four randomized controlled trials (RCTs) demonstrated that periarticular corticosteroid infiltration during TKA improved postoperative analgesia, but unlike the results of the present study, had no significant effect on PONV [10–13]. In one of these studies, patients received general anesthesia and periarticular ropivacaine infiltration with or without dexamethasone (6.6 mg) after bone cutting was completed, similar to the present study [10]. PAI with dexamethasone showed a lower incidence of PONV than PAI alone (5% vs 20%), with no significance due to the small sample size. In the other three studies, patients were anesthetized with a subarachnoid block, and periarticular ropivacaine infiltration containing adrenaline with or without a corticosteroid (methylprednisolone/triamcinolone) equivalent to 8 mg dexamethasone was performed after all bone cuts; corticosteroids did not decrease the incidence of PONV [11–13]. Since the use of a pneumatic tourniquet or surgical vacuum drain varied among these four studies, their influence on the effects of local corticosteroids remains unclear. The reason for the discrepancy between previous findings and the present results has not yet been identified; however, vasoconstriction by adrenaline included in the test solutions might have altered the effects of local corticosteroids on PONV.

The effects of perineural corticosteroid infiltration on PONV have been investigated in several RCTs. In contrast to the preceding argument, the addition of dexamethasone (8 mg) to a local anesthetic without adrenaline in patients who received a transversus abdominis block or brachial plexus block significantly decreased PONV with lower pain scores and longer analgesia postoperatively [14–18]. Among these studies, anesthesia (general or spinal anesthesia or conscious sedation) and the timing of nerve blocks (before or after surgery) varied. These findings suggest antiemetic effects of local dexamethasone infiltration and appear to support our results that periarticular dexamethasone infiltration during TKA was associated with a reduced risk of PONV.

Since the onset time of dexamethasone is considered to be approximately 2 h, the timing of dexamethasone administration to prevent PONV has been recommended immediately after the induction of anesthesia rather than at the end of surgery [19]. Nevertheless, peripheral nerve blocks with a local anesthetic combined with dexamethasone performed immediately after surgery were effective for PONV during the first 24 h postoperatively [15, 16, 18]. These findings suggest a wide range of timing for local dexamethasone infiltration to prevent PONV, and it is favorable for PAI during TKA because infiltration to the posterior periarticular tissue of the knee joint is technically limited after bone cutting at the late stage of surgery.

Although the mechanisms by which corticosteroids reduce PONV remain unclear, anti-inflammatory properties via prostaglandin antagonism are generally accepted. Dexamethasone may suppress local inflammatory responses at surgical sites and ameliorate inflammation triggered by the afferent stimulation of the parasympathetic nervous system to the vomiting center [20]. Ikeuchi et al. showed that PAI with dexamethasone decreased interleukin-6 levels in drainage fluid and serum C-reactive protein levels after TKA, suggesting the attenuation of local and systemic inflammation by periarticular dexamethasone infiltration [10]. This may have been involved in the effectiveness of dexamethasone added to PAI for PONV shown in the present study.

Another plausible mechanism for PONV prophylaxis by corticosteroids is opioid-sparing effects because the postoperative use of opioids has been identified as an independent predictor of PONV [8, 21, 22]. Perineural dexamethasone infiltration has been shown to reduce the incidence of PONV in association with a decrease in postoperative opioid consumption consistent with superior pain relief [14–16, 18]. In the present study, the multivariate analysis revealed that the addition of dexamethasone to PAI reduced the risk of PONV independently of postoperative opioid use. Further studies are needed to establish whether and to what extent opioid-sparing effects contribute to the antiemetic action of local dexamethasone infiltration.

PJI is currently the most frequently reported reason for revision in TKA [23]. Several meta-analyses of RCTs demonstrated that single periarticular corticosteroid infiltration during knee arthroplasty did not increase the incidence of wound infection [3, 4]. However, the majority of RCTs excluded patients with suboptimal glycemic control in diabetes mellitus, which has been implicated in SSI [3, 24]. In the present study, the ratio of diabetic patients was markedly lower in those who received PAI with dexamethasone, and this may have been due to the intention of surgeons to avoid the use of dexamethasone in order to prevent SSI. This selection bias may have resulted in the incidence of SSI in patients who received PAI with dexamethasone being underestimated. In addition, the duration of PJI surveillance in previous studies and ours was relatively short because the risk of PJI was the greatest within the first 2 years of TKA and remained for up to 10 years [10, 11, 13, 25, 26]. Previous studies as well as the present study were limited by the small sample size to detect extremely small differences in the incidence of PJI in TKA. Therefore, further large-scale studies with long-term observations are needed to clarify whether single periarticular corticosteroid infiltration causes PJI after TKA, particularly in diabetic patients.

The present study has several limitations. Since this was a retrospective observational study, the results obtained only revealed associations, not causal relationships between dexamethasone added to PAI and PONV prophylaxis. Furthermore, the results from a small single-center study limit the ability to generalize it to different settings. In addition, we cannot exclude the possibility of important differences in confounders influencing the relationship between dexamethasone added to PAI and PONV even after multivariable adjustments because of the possible selection bias in a retrospective design. Postoperative pain intensity, which was not directly assessed, may have confounded the present results. Furthermore, the sample size in the present study was calculated for the primary endpoint, the incidence of PONV, and was underpowered to draw a solid conclusion regarding the incidence of SSI.

## Conclusions

The results of this retrospective observational study demonstrated that dexamethasone added to PAI for postoperative pain management independently reduced the risk of PONV within 24 h of TKA, thereby supporting its addition to PAI due to increased benefits for patients in the early postoperative period. RCTs with an appropriate power to confirm the antiemetic efficacy and safety of dexamethasone added to PAI are needed in the future.

## Data Availability

The datasets analyzed during this study are available from the corresponding author on reasonable request.

## Abbreviations

ASA-PS: American Society of Anesthesiologists-physical status
CI: Confidence interval
NSAID: Non-steroidal anti-inflammatory drug
OR: Odds ratio
PAI: Periarticular anesthetic infiltration
PJI: Prosthetic joint infection
PONV: Postoperative nausea and vomiting
RCT: Randomized control trial
SSI: Surgical site infection
TKA: Total knee arthroplasty
